# Central diabetes insipidus: alert for dehydration in very low birth weight infants during the neonatal period. A case report: Diabete insípido central: alerta para desidratação no período neonatal em recém-nascidos de muito baixo peso. Um relato de caso

**DOI:** 10.1590/1516-3180.2012.6450001

**Published:** 2014-09-26

**Authors:** Maria Lúcia Silveira Ferlin, Débora Simone Sales, Fábia Pereira Martins Celini, Carlos Eduardo Martinelli

**Affiliations:** I MD, PhD. Professor, Department of Pediatrics, Faculdade de Medicina de Ribeirão Preto (FMRP), Universidade de São Paulo (USP), Ribeirão Preto, São Paulo, Brazil.; II MD, MSc. Attending Physician, Department of Pediatrics, University Hospital, Faculdade de Medicina de Ribeirão Preto (FMRP), Universidade de São Paulo (USP), Ribeirão Preto, São Paulo, Brazil.

**Keywords:** Diabetes insipidus, neurogenic, Infant, very low birth weight, Hypernatremia, Dehydration, Infant, newborn

## Abstract

**CONTEXT:**

Central diabetes insipidus (CDI) is a rare cause of hypernatremia during the neonatal period. The diagnosis is particularly difficult in very low birth weight (VLBW) newborns.

**CASE REPORT:**

We report on a preterm newborn who presented CDI soon after birth. On the third day of life, signs of dehydration were present despite normal fluid supply. The diuresis rate was 4.4 ml/kg/h. Although the fluid supply was then increased, the dehydration continued, with hypernatremia, normal glycemia, diuresis of 7.4 ml/kg/h and urine density of 1005 mOsmol/l. Thus, a diagnostic hypothesis of diabetes insipidus was raised. A test with a nasal vasopressin analogue (dDAVP) was performed and CDI was confirmed. Reduction of the fluid supply became possible through appropriate treatment.

**CONCLUSION:**

The diagnosis of CDI is rarely made during the neonatal period, especially in VLBW newborns, because of the difficulty in detecting elevated diuresis. Persistent hypernatremia, usually accompanied by hyperthermia despite abundant fluid supply, weight loss and low urine osmolality are important signs of alert.

## RESUMO

**CONTEXTO:**

Diabete insípido central (DIC) é uma rara causa de hipernatremia durante o período neonatal. O diagnóstico é difícil, particularmente em recém-nascidos (RN) de muito baixo peso (RNMBP).

**RELATO DE CASO:**

Relatamos um RN que apresentou DIC logo após o nascimento. No terceiro dia de vida, apresentava sinais de desidratação, embora estivesse recebendo aporte adequado de líquidos. A diurese aferida era de 4,4 ml/kg/h. Apesar do aumento do aporte hídrico, manteve-se desidratado, com hipernatremia, valores normais de glicemia e diurese de 7,4 ml/kg/h com densidade urinária de 1005 mOsmol/l. Desta forma, a hipótese diagnóstica de diabete insípido foi considerada. O teste com análogo da vasopressina (dDAVP) foi realizado e DIC foi confirmado. A redução do aporte de líquidos foi possível com o tratamento adequado.

**CONCLUSÃO:**

O diagnóstico de DIC raramente é realizado durante o período neonatal, particularmente em RNMBP, devido à dificuldade em detectar diurese aumentada. Hipernatremia persistente, geralmente acompanhada de hipertermia, apesar do abundante aporte de água, perda de peso e osmolaridade urinaria baixa, são importantes sinais de alerta.

## INTRODUCTION

Water and electrolyte homeostasis in newborns are influenced by postnatal physiological adaptations and appropriate management is important especially for very low birth weight (VLBW) newborns. Careful monitoring is necessary in order to maintain water and electrolyte balance. The lower the gestational age is, the higher the quantity of total and extracellular water, and consequently the greater the postnatal weight loss will be. The water and electrolyte requirements are closely correlated with physiological or abnormal losses, which are influenced by gestational and postnatal age, birth weight, ambient temperature, humidity and mechanical ventilation.^1^

Dehydration with hypernatremia can occur during the neonatal period, especially in VLBW and preterm newborns, associated with unnoticeable water loss, high urine output and reduced ability to excrete sodium load. Diabetes insipidus is a rare cause of hypernatremia during the neonatal period and it is difficult to diagnose, particularly in VLBW newborns. Persistent hypernatremia despite increased fluid intake should be an important red flag.^1^^,^^2^^,^^3^ Water homeostasis in the body is finely balanced between release of the diuretic hormone vasopressin and stimulation of thirst.

Diabetes insipidus is a disorder of water homeostasis characterized by failure to concentrate urine due to insufficient production of vasopressin (central diabetes insipidus, CDI) or due to impaired kidney response to this hormone (nephrogenic diabetes insipidus, NDI).^4^

In order to alert to the diagnosis and early treatment of CDI in VLBW newborns, in the absence of congenital infection or other comorbidities, we report here on a VLBW newborn who presented signs and symptoms of CDI soon after birth. The symptoms were properly controlled through nasal administration of 1-deamino-8-D-arginine vasopressin (dDAVP). This case report was approved by the local Ethics Committee and all authors complied with the World Medical Association’s Declaration of Helsinki regarding ethical conduct of research involving human subjects.

## CASE REPORT

A male preterm newborn with gestational age of 31 weeks, birth weight of 1180 g (5^th^ percentile) and Apgar scores of 2 and 3 at the 1^st^ and 5^th^ minutes, respectively, was born by means of vaginal delivery with pelvic presentation. After 21 hours, the baby was transferred to the University Hospital of the Ribeirão Preto School of Medicine (Faculdade de Medicina de Ribeirão Preto, FMRP), hyaline membrane disease was diagnosed and nasal continuous positive airway pressure (CPAP) was instituted. The serum sodium, potassium and calcium levels were 146 mEq/l, 5.8 mEq/l and 7.9 mg/%, respectively. On the third day of life, he presented dehydration despite a fluid supply of 170 ml/kg/day. On that day, the diuresis rate was 4.4 ml/kg/hour, with no glycosuria. Glycemia was 160 mg/% and the serum sodium concentration was 149 mEq/l. No abnormalities were observed regarding serum potassium and calcium levels.

The patient was then positioned in an incubator in order to prevent additional dehydration. However, the patient remained dehydrated with hypernatremia despite an increase in the fluid supply to 300 ml/kg/day (two thirds orally and one third intravenously). The urine output ranged from 2.4 to 7.4 ml/kg/h and the urine density was around 1005 mOsmol/l. The hypothesis of diabetes insipidus was raised and a therapeutic test with dDAVP confirmed the diagnosis of CDI, ruled out the possibility of NDI and allowed a reduction of the water supply to 150 ml/kg/day (Table 1).

**Table 1.**
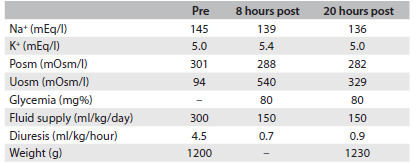
Serum sodium (Na^+^) and potassium (K^+^) concentrations, plasma osmolality (Posm), urinary osmolality (Uosm), glycemia (mg%), fluid supply (ml/kg/day), diuresis (ml/kg/hour) and weight (g) before (pre) and 8 and 20 hours after (post) administration of 0.012 ml dDAVP (1-deamino-8-D-arginine vasopressin)

100 µg = 1 ml of nasal solution of dDAVP.

Transfontanelle neurosonography performed at 18 days of life showed slight hydrocephaly and hematoma compatible with grade III intracranial hemorrhage. Evaluation of the remaining pituitary axes revealed normal thyroid, adrenal and gonadal function.

Serological tests for syphilis, toxoplasmosis, cytomegalovirus (CMV) and rubella ruled out these congenital infections. Negative results also came from blood and cerebrospinal fluid cultures for bacterial agents.

The patient was discharged from the hospital at 3.5 months of age, weighing 3560 g, with a prescription that comprised 0.01 ml of intranasal dDAVP every 12 hours and a recommendation of fluid intake of 180 ml/kg/day.

Magnetic resonance imaging (MRI) of the brain performed at five months of age revealed a pituitary gland of height 4 mm and absence of hyperintense signal from the neurohypophysis.

At five months of corrected gestational age (CGA), he presented deceleration of weight-height gain, remaining below the 10^th^ percentile until reaching two years of CGA, despite appropriate nutrition and treatment with intranasal dDAVP every 12 hours. Transfontanelle neurosonography performed at one year of age was normal.

## DISCUSSION

The diagnosis of CDI is rarely made during the neonatal period, especially in VLBW newborns. Persistent hypernatremia, usually accompanied by hyperthermia despite a large fluid supply, weight loss and low urine osmolality are important signs of alert, considering the difficulty in detecting an elevated urine output.^5^

The main controller of urine output is vasopressin (AVP), which is released by the posterior pituitary and increases free water absorption by the kidney. AVP is a peptide of nine amino acids that is produced by magnocellular neurons of the supraoptic and paraventricular neurons of the hypothalamus. Its synthesis shares a common precursor with neurophysin and its secretion is closely related to that of neurophysin, to which it remains bound until it is secreted into the bloodstream.^6^

Excessive production of diluted urine can be secondary to three main causes: excessive administration of fluid, reduced AVP production (CDI) or reduced renal response to this hormone (NDI). In the patient reported here, the findings of dehydration with polyuria, hypotonic urine, hypernatremia and high plasma osmolality enabled the diagnosis of diabetes insipidus and the good response to treatment with the synthetic vasopressin analogue dDAVP ruled out the diagnosis of NDI, which is a more common etiology for diabetes insipidus within this age range. The clinical manifestations of congenital CDI usually occur later, as the result of gradual degeneration of the AVP-producing neurons due to intracellular accumulation of the AVP-neurophysin complex.^6^

Neonatal CDI has been described as a complication of intrauterine and perinatal diseases; therefore, neuroimaging can be of help in identifying possible etiologies. Possible causes that may be considered include: asphyxia, severe infections, congenital infections, peri or intraventricular hemorrhage and CNS abnormalities.^7^^,^^8^

Although the etiology and treatment of CDI has been described previously in several case series among children, it remains a relatively rare disorder in the neonatal population, and specifically in VLBW newborns. The importance of this report is to alert neonatologists regarding this possible diagnosis when they are faced with cases of dehydration and hypernatremia in the neonatal period, given that this is not among the most common causes. Early recognition of the disorder and its proper management may be the key to patient survival.

Although the patient reported here was premature, he did not present congenital infection, central nervous system (CNS) abnormalities or severe anoxia that might have explained the CDI. The early onset of signs and symptoms also ruled out the possibility of congenital CDI, and therefore intracranial hemorrhage became the most likely etiology. Neonatal CDI is usually life-threatening and requires a high degree of suspicion, and not many cases without congenital infections or CNS abnormalities/malformations have been described in the literature^2^^,^^3^^,^^9^^,^^10^^,^^11^^,^^12^

MRI performed at five months of age showed a pituitary gland with a height of 4 mm and absence of the normal hyperintense signal from the neurohypophysis, a finding that is highly suggestive of CDI. Although absence of a hyperintense signal corresponding to the posterior pituitary on MRI is a frequent finding in CDI, evidence of a hyperintense signal does not necessarily indicate integrity of the system, as observed in some autosomal dominant and idiopathic forms.^13^

A previous case report on a premature infant with CDI secondary to intracranial hemorrhage had a transient course.^14^ That case may have been due to cerebral edema.^15^ CDI is not necessarily transient in nature, but its duration depends on the location of the damage in the hypothalamic-pituitary region.^3^^,^^9^^,^^10^ MRI of the brain should be performed later in infancy if CDI persists.^16^ In our case, CDI was persistent and it was necessary to maintain administration of intranasal dDAVP. The neurosonography performed at one year of age was normal.

AVP replacement in the form of desmopressin is the treatment of choice for CDI and special care must be taken to avoid fluid overload in small babies. Oral administration has been recommended by some authors, although properly performed intranasal application can be a quite safe option. A search regarding the topic of central diabetes insipidus in very low birth weight infants during the neonatal period was made in Medline (Medical Literature Analysis and Retrieval System Online), Lilacs (Literatura Latino Americana e do Caribe em Ciências da Saúde) and IBECS (Índice Bibliográfico Espanhol em Ciências da Saúde) (Table 2).

**Table 2.**
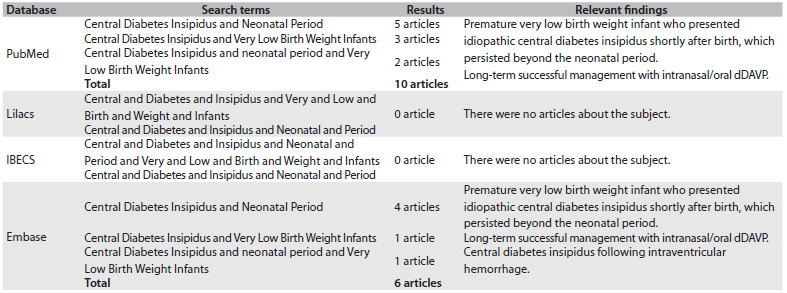
Search strategies performed on August 28, 2013, and results from Medline (Medical Literature Analysis and Retrieval System Online), Lilacs (Literatura Latino Americana e do Caribe em Ciências da Saúde) and IBECS (Índice Bibliográfico Espanhol em Ciências da Saúde)

dDAVP = 1-deamino-8-D-arginine vasopressin.

## CONCLUSIONS

Diagnosing diabetes insipidus during the neonatal period is difficult and its etiology often remains unknown. The combination of weight loss, hypernatremia, increased diuresis, low urinary osmolality and high plasma osmolality should evoke the diagnosis of diabetes insipidus even in the absence of congenital or acquired infection.
